# Quality of antenatal care services and completion of four or more antenatal care visits in Ethiopia: a finding based on a demographic and health survey

**DOI:** 10.1186/s12884-017-1488-0

**Published:** 2017-09-11

**Authors:** Kindie Fentahun Muchie

**Affiliations:** Epidemiology and Biostatistics Department, Institute of Public Health, College of Medicine and Health Sciences, University of Gondar, Gondar, Ethiopia

**Keywords:** ANC, Four or more visits, Determinants, Clustered data, Ethiopia

## Abstract

**Background:**

Antenatal care (ANC) is one of the core interventions for improving maternal outcomes. The average annual decline of maternal mortality rate from 1990 to 2013 was 5% in Ethiopia. This figure was below the least expected 5.5% to achieve the targeted 75% by 1990–2015. Moreover, completion of the recommended four or more ANC visits was only 32%. This study was aimed to examine individual, household and community level potential determinants of completing the recommended visits in the country.

**Methods:**

The 2014 Ethiopian Mini Demographic and Health Survey data were used. Among women aged 15–49 years 3694 who had given birth in the 5 years preceding the survey were included in the analysis. The robust standard error method of generalized estimation equations were used for binary outcome variable from the clustered data.

**Results:**

Only 33.0% (95% CI 31. 5% 34.5%) of women completed the recommended visits. Out of the total women, 56.5% had at least one ANC visit. Out of those who had at least one ANC visit, 37.4% visited in their first trimester. Completing the recommended visits was negatively associated with women in the lower educational level, lower economic conditions, higher birth order, and rural residence. But, it was positively associated with the community level high quality ANC services received. Difference in age and region also affected the completion of the recommended visits.

**Conclusion:**

The finding revealed the need for improving the uptake of ANC services, early arrival in the first trimester for services, and motivating mothers that begin ANC to confirm continuity. Strategies to foster completing the recommended visits should focus on upgrading quality of care services at the community level. Women in low economic level, high birth order, rural residence, and low educational status should be given special attention. Early and late age groups should be given special attention regarding the services.

## Background

Antenatal care (ANC) is one of the core interventions for improving maternal outcomes [1]. ANC services enable early identification of pregnancy related risks and complications; and ensure access of services including health education, vaccines, diagnostic tests and treatments [2]. It also helps to establish good relationship between pregnant women and service providers [3]. Moreover, a pregnant woman visiting health facilities for ANC would get advice and support, and will be more informed about health needs and self-care [4], and consequently leaded to an increased utilization of emergency care services [5, 6].

In Ethiopia the average annual decline of maternal mortality rate was 5% from 1990 to 2013 [7]. This figure was below the least expected 5.5% to achieve the targeted 75% decline between 1990 and 2015 [7]. Out of 289,000 estimated maternal death globally in 2013, 62% were from the Sub-Saharan Africa region alone [7]. In the same 2013 report, Ethiopia was one among ten countries comprising 58% of the global maternal deaths with 420 deaths per 100,000 live births.

According to the WHO report, 83% of pregnant women received ANC at least once in the period 2007–2014 globally. However, in the same report 64% had the recommended four or more ANC visits [8]. In Ethiopia ANC services for women are provided at community health post, health center and hospital. However, in this country, 58% and 32% of the women with live births in the 5 years before the survey had at least one and four or more ANC visits, respectively for services from skilled health personnel [9]. Although the Ethiopian Federal Ministry of Health has been working to improve maternal outcomes, the figures show improving access to and strengthening facility based maternal services is still needed.

Several studies conducted among specific localized areas in Ethiopia examined factors affecting ANC utilization [10–13], but these were neither representative of the country nor particular about the recommended minimum of four visits. The studies considered only individual and household characteristics associated with the utilization of ANC services. However, literatures suggested that context can influence individual health outcomes [14, 15]. Communities are local contexts and specific localities where individuals reside, share physical and socioeconomic characteristics, and establish social networks that potentially affect individual health and explain the impact of social inequalities in accessing health services [14].

This paper attempted to assess individual, household and community related determinants of completing four or more ANC visits among women of reproductive age in Ethiopia by considering the clustered nature of the data. In order to address this objective, recent data from a large-scale household survey conducted in 2014 provided a valuable opportunity. The analysis of determinants allowed a better identification of women who didn’t utilize ANC services four or more times with a high probability; as a result a more effective and efficient application of interventions was secured.

## Methods

### Data and variables

The 2014 Ethiopian Mini Demographic and Health Survey (EMDHS) data set was used for this analysis. The survey designed to obtain and provide information on basic indicators, including ANC visits, utilized multi stage sampling technique. The study population comprised of women in Ethiopia aged 15 to 49 years and gave birth at least once in the last 5 years preceding the survey. Out of 8070 individual women surveyed, data on the last live birth for 3694 women with complete information on variables considered under the study were used. The binary response variable considered in this study indicated whether women completed four or more ANC visits or not from skilled health personnel including Doctors, Nurses, Mid-wives and/or Health officer. Selection of several potential factors associated with completing four or more ANC visits was based on related studies conducted on the factors influencing ANC utilization [16–21]. Explanatory variables like current age, educational status, residence, region, marital status, sex of household head, birth order, and wealth index were used at individual and household levels. The wealth index variable in the EMDHS included was obtained by using principal component analysis with five quintiles. However, the two low (lowest and lower taken as low) and the upper most two (highest and higher taken as high) categories were merged for the purpose of this analysis. Two categorical variables derived from a self-exclusive proportion were considered, of which the first was a self-exclusive proportion of educated women, showing the community level literacy of women. The second was a self-exclusive proportion of women who took five components of ANC services, showing community related quality of ANC services received [20, 21]. A binary indicator for quality of care was created, distinguishing between proportions less or equal to 5% (low) and proportions more than 5% (high). The five components considered were all of the six components of ANC which the WHO has determined essential for every pregnant woman except weight measurement. Accordingly, the five components considered were measuring blood pressure, taking sample of blood, taking sample of urine, giving iron tablets/syrup, and informing about pregnancy complications. For both the community related variables cut-off points were chosen based on the distribution of cases. Cut-off points were specifically chosen on the basis that could not violate assumptions of the model (cell counts in the covariate pattern used directly for measuring goodness of fit), could enable us to get not small number of categories (important properties for different proportions), and cut-off points were tried to be better practical values (like products of 5, 10, 25, and/or 100).

### Statistical analysis

The Statistical software Stata version 11 was used for the whole analysis. Due to the similarity of health facility centers, training, and the similar life style conditions, it was expected that responses from the same enumeration area would behave more alike than those of different enumeration areas. As a result, respondent women were clustered within an enumeration area. Thus, the standard error estimates using independent observations was no longer proper for data sets with cluster structures. This analysis considered the intra-cluster correlation and used the Huber method of robust standard error estimates among the family of generalized estimation equations for clustered data in the binary logistic regression modeling [22, 23].

## Results

### Characteristics of the sample

Among the 3694 sampled women, only 33.0% (95% CI 31. 5% 34.5%) completed four or more ANC visits (Table 1). Out of the total women considered for this study 56.5% (95% CI 54.8% 58.1%) had at least one ANC visit. Besides, out of those who had at least one ANC, 37.4% (95% CI 35.3% 39.5%) visited in their first trimester.

**Table 1:** Distribution of completing four or more antenatal care visits by categories of selected variables among women in Ethiopia, 2014. (*N* = 3694)

Variables	Categories	Count (%)	% of 4 or more ANC visits
Marital status	Never in union/Formerly in union	281 (7.61)	39.86
	Currently in union	3413 (92.39)	32.43
Birth Order	1	691 (18.71)	48.19
	2 or 3	1105 (29.91)	38.10
	4 or 5	922 (24.96)	27.01
	6 +	976 (26.42)	22.13
Community level Quality of received ANC	Low	1902 (54.34)	25.55
	High	1598 (45.66)	45.87
Community level literacy	Low	913 (24.72)	14.79
	Medium	1932 (52.30)	29.35
	High	849 (22.98)	60.90
Wealth index	Poor	1832 (49.59)	16.7
	Medium	572 (15.48)	31.99
	Rich	1290 (34.92)	56.59
Educational status	No education	2368 (64.1)	21.11
	Primary	1006(27.23)	45.63
	Secondary/higher	320(8.66)	81.25
Place of residence	Urban	740 (20.03)	71.62
	Rural	2954 (79.97)	23.32
Region	Tigray	309 (8.36)	40.13
	Afar	369 (9.99)	11.92
	Amhara	386 (10.45)	22.28
	Oromia	460 (12.45)	32.17
	Somali	347 (9.39)	5.48
	B. Gumuz	306 (8.28)	27.78
	SNNP	512 (13.86)	31.64
	Gambela	302 (8.18)	33.44
	Harari	256 (6.93)	46.88
	Addis Ababa	177 (4.79)	91.53
	Dire Dawa	270 (7.31)	62.22
Age	15–19	190 (5.14)	24.21
	20–24	696 (18.84)	35.92
	25–29	1123 (30.40)	35.26
	30–34	777 (21.03)	35.91
	35–39	575 (15.57)	31.48
	40–44	228 (6.17)	19.30
	45–49	105 (2.84)	21.9
HH Sex	Male	3147 (85.19)	31.87
	Female	547 (14.81)	33.0

The result also showed that out of samples taken more than three fourths (80.0%) were rural residents. Only 23.3% of the rural residents completed four or more ANC visits, while 71.6% of the urban residents completed the recommended four or more ANC visits.

Based on the community level quality of received ANC, 54.3% women lived in a community with a low level quality of received ANC, while 45.7% lived in a community with high community level quality of received ANC. Besides, 45.9% of women living in a community with high quality of received ANC completed four or more ANC visits, whereas only 25.6% of those in a community with low quality of received ANC completed the four or more ANC visits. With respect to community level literacy, 24.7% of women lived in a community with low community level literacy of women. This figure was 52.3% and 23.0% for women in the community with medium and high community level literacy of women, respectively. Moreover, 60.9% of the women in the community which had a high level literacy of women completed four or more ANC visits, while the figures were 29.4% and 14.8% for women in low and medium community level literacy of women, respectively.

When considered from the point of view of wealth index, almost half (49.6%) of the women who took part in the study were from poor households, While 15.5% and 34.9% were from medium and rich ones, respectively. Among women who were from poor, medium and rich households, 16.7%, 32.0% and 56.6% completed four or more ANC visits, respectively. At individual levels, 18.7% of the women with their last delivery were first birth order pregnancies. This figure was 29.9%, 25.0%, and 26.4% for 2nd or 3rd, 4th or 5th and above 6th birth orders, respectively. The proportion of those who completed four or more ANC visits decreased as the birth order increased. For instance, 48.2% completed four or more ANC visits among first birth order pregnancies of their last delivery.

### Determinants of completing the four or more ANC visits

The result of the bi-variable analysis showed that all explanatory variables were associated with completing four or more ANC visits at 25% level of significance. The final adjusted model, including all considered factors fits the data well (Goodness of fit test *p*-value = 0.3516). This could also be supported by area under Receiver Operating Curve (ROC) with a value of 0.8044.

The result showed that variables such as age and highest level of education of women, region, residence, and wealth index of the household; birth order of the child and community level quality of received ANC services were significant determinants of completing four or more ANC visits at 5% level of significance (Table 2).

**Table 2:** Bi-variable and multi-variable odds ratios for potential factors of completing four or more ANC visits in Ethiopia, 2014

Variables	Categories	COR[95% CI]	AOR[95% CI]
Marital status	Never in union/Formerly in union	1	1
	Currently in union	0.72[0.56, 0.94]	0.97[0.68, 1.39]
Birth Order	1st	1	1
	2nd or 3rd	0.66[0.54, 0.81]	0.79[0.60, 1.05]
	4th or 5th	0.40[0.31, 0.52]	0.62[0.44, 0.88]
	6th +	0.30[0.23, 0.40]	0.64[0.42, 0.96]
Community level Quality of received ANC	Low	1	1
	High	2.47[1.84, 3.30]	1.42[1.03, 1.96]
Community level literacy	Low	1	1
	Medium	2.39[1.62, 3.52]	1.08[0.75, 1.54]
	High	8.97[5.80, 13.89]	1.14[0.73, 1.79]
Wealth index	Poor	1	1
	Medium	2.34[1.85, 2.97]	1.78[1.41, 2.24]
	Rich	6.50[1.94, 2.55]	1.84[1.40, 2.40]
Educational status	No education	1	1
	Primary	3.13[2.61, 3.77]	1.86[1.56, 2.21]
	Secondary/ Higher	16.19[10.52, 24.92]	3.67[2.53, 5.31]
Place of residence	Urban	1	1
	Rural	0.12[0.08, 0.18]	0.35[0.22, 0.53]
Region	Tigray	1	1
	Afar	0.20[0.09, 0.44]	0.33[0.19, 0.58]
	Amhara	0.43[0.254, 0.73]	0.56[0.34, 0.94]
	Oromiya	0.71[0.41, 1.20]	0.727[0.44, 1.19]
	Somali	0.09[0.04, 0.21]	0.15[0.07, 0.32]
	B. Gumuz	0.57[0.28, 1.18]	0.78[0.37, 1.63]
	SNNP	0.69[0.39, 1.23]	0.87[0.47, 1.60]
	Gambela	0.75[0.41, 1.36]	0.70[0.39, 1.26]
	Harari	0.1.31[0.66, 2.64]	0.59[0.34, 1.01]
	Addis Ababa	16.11[8.37, 31.00]	2.56[1.32, 4.93]
	Dire Dawa	2.46[1.25, 4.85]	1.82[0.94, 3.50]
Age	15–19	1	1
	20–24	1.75[1.21, 2.55]	1.71[1.12, 2.62]
	25–29	1.71[1.16, 2.51]	2.20[1.36, 3.56]
	30–34	1. 75[1.19, 2.59]	2.67[1.63, 4.40]
	35–39	1.44[0.95, 2.17]	2.35[1.35, 4.07]
	40–44	0.75[0.47, 1.19]	1.52[0.82, 2.78]
	45–49	0.87[0.50, 1.55]	1.864[0.94, 3.70]
HH Sex	Male	1	1
	Female	1.39[1.13, 1.73]	1.11[0.84, 1.46]

*COR* crude odds ratio, *AOR* adjusted odds ratio, *CI* confidence interval

The odds of completing four or more ANC visits for women aged 20–24, 25–30, 30–34, and 35–39 years were 1.71 [OR 1.71, (95% CI 1.12, 2.62)], 2.20 [OR 2.20, (95% CI 1.36, 3.56)], 2.67 [OR 2.67, (95% CI 1.63, 4.40)], and 2.35 [OR 2.35, (95% CI 1.35, 4.07)], times that for women aged 15–19 years, respectively.

Women with primary, and secondary/higher educational levels were 1.86 [OR 1.86, (95% CI 1.56, 2.21)], and 3.67 [OR 3.67, (95% CI: 2.53, 5.31)] times likely to complete four or more ANC visits as compared to un-educated women, respectively.

The result revealed that rural dweller women were 65% [OR 0.35, (95% CI 0.22, 0.53)] less likely to complete four or more ANC visits as compared to urban ones. On the basis of economic status, women in the medium, and rich households were 78% [OR 1.78, (95% CI 1.41, 2.25)], and 84% [OR 1.84, (95% CI 1.41, 2.40)] more likely to complete four or more ANC visits, respectively as compared to their counter parts.

Fourth/fifth, and sixth/above birth order pregnant women were 38% [OR 0.62, (95% CI 0.44, 0.88)], and 36% [OR 0.64, (95% CI 0.42, 0.96)] less likely to complete four or more ANC visits, respectively as compared to 1st birth order pregnant women.

Women in communities with high quality of received ANC were 42% [OR 1.42, (95% CI 1.03, 1.96)] more likely to complete four or more ANC visits as compared to women in communities with low quality of received ANC.

Women in Somali, Afar, and Amhara Regional States were respectively 85% [OR 0.15, (95% CI 0.07, 0.32)], 67% [OR 0.33, (95% CI 0.19, 0.58)] and 44% [OR 0.56, (95% CI 0.34, 0.94)] less likely, while those in Addis Ababa City Administration were 2.56 [OR 2.56, (95% CI 1.32, 4.93)] times likely to complete four or more ANC visits as compared to women in Tigray Regional State.

## Discussion

In this study, individual, household, and community level factors were considered as determinants of completing four or more ANC visits in Ethiopia. This study differed from others carried out in Ethiopia [10–13] to date in that it considered community related variables and used binary logistic regression model with robust standard error estimates. This analysis was helpful to quantify the association of community related variables with completion of four or more ANC visits so as to design more effective policies, define appropriate intervention and reduce community based inequalities in accessing ANC services.

Candidate predictors of the multivariable binary logistic regression model were those significantly associated with completing four or more ANC visits from the bi-variable analysis at 25% level of significance. From the final multivariable model, the variables age and highest level of education of the woman, region, residence and wealth index of the household; birth order of the child and community level quality of received ANC were related with completing the four or more ANC visits in Ethiopia.

The result showed that as women’s educational level increases the likelihood of completing the four or more ANC visits also increases. This result agreed with those of studies conducted abroad, in Vietnam [16], Colombia [19], and Brussels Metropolitan region [18]. This result could be due to the fact that education is an indicator of various other factors associated with health seeking behavior. It could be reasonable to say that educated women as compared to uneducated, have better access to information, possess a level of health literacy that could empower them to exercise their choice, and able to overcome cultural barriers of ANC service utilization [24, 25]. Furthermore, education changes attitude and expectation of a woman and her significant others towards traditional gender norms and roles [26]. Lack of education leads to poor quality interactions between a pregnant and service providers consequently discouraging utilization of ANC services [27]. The study suggested that mothers in Ethiopia should be educated about ANC utilization to raise uptake of services at health facilities [28]. The study also showed the need of strengthening interventions mitigating early drop out of girls from school.

The result also indicated that the likelihood of completing four or more ANC visits decreased as birth order increased as reported consistently by studies in Haiti [20], Nigeria [29], and Brussels Metropolitan region [18]. This result implies that high parity women have less desire to use recommended ANC visits. This could be due to the belief that they do not need services as they have experience with pregnancy and childbirth. Besides, structural barriers related to cost and time raised from higher child dependency ratio prevents seeking ANC services for higher parity women.

Moreover, at individual levels, women aged 20–39 years were more likely to complete the four or more ANC visits as compared to those aged 15–19 years. This finding was consistent with the study conducted in Colombia [19].

Women from Somali, Afar and Amhara Regional States had less chance from severe to slight, respectively to complete the four or more ANC visits. This could be due to the fact that in availability of access to health services, lower economic status, lack of political commitment, hostile and isolated environments, and large number of ethnic minorities in majority of areas of these regions [30, 31].

Rural resident women were less likely to complete the four or more ANC visits as compared to urban dwellers in Ethiopia. Other studies in Nepal [32] and Vietnam [33] found out results consistent with ours.

The likelihood of completing four or more ANC visits increased as household economic status rose. This result was consistent with those of studies in Vietnam [16], Haiti [34], Nigeria [24], and Kenya [35]. Poverty is a structural factor that could affect a woman’s ability to seek ANC through multiple mechanisms, including ability to identify and locate the availability of services, overcoming financial challenges, with standing geographic inaccessibility, possessing knowledge about services, improving attitudes towards services, and improving provider attitudes [36].

Women in the community that received high quality of ANC were more likely to complete four or more ANC visits. This result was consistent with that of a study in Zambia [21] that showed the odds of having three or fewer ANC visits were higher if the quality of ANC received was lower. A related study in Haiti [20] showed that high-quality ANC was associated with increased odds of using skilled birth attendance even after controlling for the number of ANC visits. The number of essential components of care received would show the quality of ANC. In our study these included whether a woman took iron tablets, was informed about the signs of pregnancy complications, and received screening tests like blood pressure measurement, urine sample and blood sample taking during ANC visits.

### Limitations

Some social and community level variables, including neighborhood security/safety, dominant ethnic group and religion were not included as they were not captured in the secondary data used for this study. Besides, causality could not be established as the study was derived from cross sectional survey.

## Conclusion

The finding highlighted the need to improve the uptake of ANC services, early arrival in the first trimester for ANC services, and motivating mothers that begin ANC to confirm continuity.

Strategies to foster completing four or more ANC visits should focus on upgrading quality of care services at community level. Moreover, women in low economic status, high birth order, rural residence, and low educational level should be given special attention. Early age group and late age group among fertile age group women should be given special attention for the services. Women from Somali, Afar, and Amhara Regions should be given special emphasis.

## Abbreviations

ANC: Antenatal care
AOR: Adjusted Odds Ratio
CI: Confidence Interval
COR: Crude Odds Ratio
EMDHS: Ethiopian Mini Demographic and Health Survey
OR: Odds Ratio
ROC: Receiver Operating Curve
WHO: world health organization
